# Evaluation of dry eye symptoms and risk factors among medical students in Serbia

**DOI:** 10.1371/journal.pone.0275624

**Published:** 2022-10-24

**Authors:** Luna Aćimović, Svetlana Stanojlović, Tanja Kalezić, Bojana Dačić Krnjaja

**Affiliations:** 1 Clinical Hospital Center Zemun, Belgrade, Serbia; 2 Faculty of Medicine, University of Belgrade, Belgrade, Serbia; 3 Clinic for Eye Diseases, University Clinical Center of Serbia, Belgrade, Serbia; Universidad de Murcia, SPAIN

## Abstract

**Background:**

Dry eye is a multifactorial disease defined less than 30 years ago. It is a relatively common disorder, affected by a number of well-known risk factors. Dry eye can be challenging to diagnose because of the possible discrepancy between patients’ symptoms and clinical signs, and its overlap with other ocular surface diseases. Literature-wise, dry eye is usually associated with age and therefore investigated within older populations. Recently, studies focusing on young adult and student populations have demonstrated a higher prevalence of dry eye than previously expected.

**Aim:**

The study aims to determine the frequency of dry eye symptoms in the student population, and the impact of students’ activities and habits as potential risk factors.

**Methodology:**

Our study involved 397 students from the medical school at the University of Belgrade, Serbia. Students were asked to complete an online survey that addressed general information, health, habits, and routine in everyday use of electronic devices. In addition, students completed a standard Ocular Surface Disease Index questionnaire.

**Results:**

The prevalence of dry eye was 60.5% (240/397) in our study population. Contact lens wear (p<0.001), allergies (p = 0.049) and increased number of hours per day using VD devices for studying purposes (p = 0.014) were associtated with a higher risk of dry eye disease. Risk factors that did not significantly impact dry eye were the use of oral contraceptives, smoking, systemic diseases, year of study and sex.

**Conclusion:**

In our study, the prevalence of dry eye disease was similar or slightly higher than in previous studies among young adults. In addition, contact lenses, allergies and visual display devices were associated with the development of the dry eye.

## Introduction

Dry eye disease (DED) is a common tear film disorder that has been documented worldwide in all age groups and ethnicities. It was recognized as a disorder less than 30 years ago, with progress being made in understanding the basis and impact of this disease. It is one of the most common reasons for patient referral to an ophthalmologist [1].

Dry eye is a multifactorial disease of the ocular surface. It is characterized by a loss of homeostasis of the tear film and accompanied by ocular symptoms, in which tear film instability and hyperosmolarity, ocular surface inflammation and damage, and neurosensory abnormalities play etiological roles [2]. Symptoms of DED include eye dryness, ocular pain, a burning sensation, visual problems, eye fatigue, sensitivity to light and itchy eyes [3–5]. The definition of the DED through symptoms encompasses all stages of the disease, including the initial stages when evident signs of disease are still nonexistent. Therefore, assessing individual patients through questionnaires would give a better insight into morbidity. In addition to the Ocular Surface Disease Index (OSDI), a reliable and validated method for measuring the severity of DED and its influence on visual function [6], numerous other tools are used to evaluate the symptoms and their impact on every day life (Impact of dry eye on everyday life questionnaire, Dry Eye-Related Quality-Of-Life Score, Dry Eye Questionnaire, and the Standard Patient Evaluation of Eye Dryness Questionnaire). Friedman reported that mild to severe DED impacts the quality of life to a similar degree as mild to severe angina when examined using utility assessment questionnaires [7].

The prevalence of DED ranges from approximately 5% to 50% [8]. It differs according to the definition, classification, diagnostic criteria, and population of interest. DED has been shown to be more prevalent among women and the elderly [9, 10]. However, several recent studies investigated DED in the young adult population, and reported a prevalence up to 70.8% [11–15]. These findings indicate the possibility of a higher prevalence of DED in this age group than previously thought. Most of these studies were conducted in Asia, South America, and Africa; to the best of our knowledge, there are not many studies on this topic amongst the European population.

In addition to age and female sex, significant risk factors include Asian race, Meibomian gland dysfunction, connective tissue diseases, Sjögren’s syndrome, computer use, contact lens wear, androgen deficiency, environmental factors, hormone replacement therapy, hematopoietic stem cell transplantation, use of medications (antihistamines, antidepressants, anxiolytics, isotretinoin), and allergies [8]. Aside from these, already established factors, there is also the recently reported problem of increased daily use of visual display (VD) devices, especially in young adults [16]. Nowadays, young generations are exposed to VD devices from a very young age. Moreover, this habit could be even more pronounced after the Covid-19 pandemic, where most young adults had to study and socialize online more than in previous years. Therefore, it is of great significance to determine the prevalence of DED and its risk factors in the young adult population for better prevention and diagnosis of DED.

The aim of this study was to determine the prevalence of DED and its risk factors among medical students at the University of Belgrade, Serbia.

## Materials and methods

In total, 397 students attending the School of Medicine, University of Belgrade, Serbia, participated in this cross-sectional study. The median age of this population was 22.5 years (range, 18–34 years). The survey was conducted in August and September 2019. Students were asked to complete an anonymous electronic survey which comprised of questions divided into three sections. The link to the questionnaire was attached to the school’s website. This study was performed in accordance with The Declaration of Helsinki. Informed consent was obtained from all participants by ticking the relevant box before beginning the survey.

In the first part of the questionnaire, students were asked to give general information: sex, age, and the year of study that they were attending, followed by questions about their health (allergies and systemic diseases) and habits (contact lens wear, smoking, use of contraceptive pills) (S1 Fig). The second part comprised questions concerning their studying habits and everyday use of computers, mobile phones, tablets, and similar digital devices, as well as their use during their free time (S1 Fig). The questions were as follows: (1) How many hours per day do you study? (2) How many hours per day do you study using hard copy sources of literature? (3) How many hours per day do you study from computers, laptops, and other digital devices? (4) How many hours per day do you use a mobile phone, computer, laptop, or tablet when you are not studying? For every question, students were asked to choose the answer: a) I do not use, b) 0–3 h per day, c) 3–6 h per day, and d) more than 6 h per day.

The third part was the standard OSDI questionnaire (S2 Fig). The total OSDI score was calculated for every student based on the formula: the sum of scores for all answered questions multiplied by 25, divided by the total number of answered questions. Values of the OSDI score range between 0 and 100, where a higher score indicates a more severe DED (S2 Fig). In this study, the cut-off for symptomatic DED was an OSDI score greater than or equal to 13.

After analyzing the descriptive statistics, we established the OSDI score for each student using the OSDI formula. According to our cut-off value, we divided them into two groups. Students who had a score greater than or equal to 13 were recognized as having symptomatic DED, and those who scored less than 13 did not have DED. We compared these two groups according to each factor included in the first part of the questionnaire (S1 Fig). Finally, the number of hours of electronic device use across different purposes was compared in the symptomatic and asymptomatic students.

### Statistical analysis

SPSS software (version 20.0, IBM, Armonk, NY, USA) was used for statistical analysis. Frequency, mean, and standard deviation were used for descriptive statistics. For the comparison of categorical variables between groups chi-square test was used. A logistic regression was performed to ascertain the effects of different risk factors on the likelihood that participants have DED. The odds ratio (OR) and 95% confidence intervals (CI) were calculated. The level of significance for all statistical analyses was set at p<0.05.

## Results

A total of 397 participants were included in this study, with an average age of 22.6 years ± 2.3 (range:18–34 years). The majority of the population were women and were not contact lens users (Table 1). Further characteristics are displayed in Table 1.

**Table 1:** Epidemiological characteristics in the medical students population.

		N	Percentage %
**Sex**	Male	96	24.2%
	Female	301	75.8%
**Contact lens use**	Yes	82	20.7%
	No	315	79.3%
**Allergies**	Yes	130	32.7%
	No	267	67.3%
**Systemic diseases**	Yes	15	3.8%
	No	382	96.2%
**Smoking**	Yes	90	22.7%
	No	307	77.3%
**Contraceptive pills**	Yes	27	6.8%
	No	370	93.2%

The OSDI score for each student was determined through the OSDI questionnaire. The average OSDI score for the examined population was 20.6 ± 16.5. After analyzing the data, 60.5% of the participants had a symptomatic DED (240 students). The majority of students (45.4%, n = 109) had a mild form of DED, while 20.8% (n = 50) had a moderate presentation, and 33.8% (n = 81) had a severe form. The prevalence of symptomatic DED among female and male respondents was 62.8% and 53.1%, respectively (Table 2). The differences in prevalence between sexes and age were not statistically significant (p = 0.092 and p = 0.283, respectively). Contact lens users were significantly more often affected by DED symptoms (p<0.001). Other possible factors that were analyzed had no significant relationship with DED (see Table 2).

**Table 2:** General information of the students with and without DED symptoms.

		Dry eye (OSDI≥ 13)				
		No N = 157		Yes N = 240		
		N	%	N	%	P-value
**Sex**	Male	45	46.9%	51	53.1%	0.092
	Female	112	37.2%	189	62.8%	
**Year of study**	First	29	49.2%	30	50.8%	0.367
	Second	30	41.1%	43	58.9%	
	Third	32	42.7%	43	57.3%	
	Fourth	11	29.7%	26	70.3%	
	Fifth	14	31.8%	30	68.2%	
	Sixth	41	37.6%	68	62.4%	
**Contact lens use**	Yes	16	19.5%	66	80.5%	**<0.001**
	No	141	44.8%	174	55.2%	
**Allergies**	Yes	43	33.1%	87	66.9%	0.066
	No	114	42.7%	153	57.3%	
**Systemic diseases**	Yes	6	40.0%	9	60.0%	0.971
	No	151	39.5%	231	60.5%	
**Smoking**	Yes	30	33.3%	60	66.7%	0.170
	No	127	41.4%	180	58.6%	
**Contraceptive pills**	Yes	11	40.7%	16	59.3%	0.895
	No	146	39.5%	224	60.5%	

A Chi-square test was performed, p<0.05 was considered as statistically significant.

Students’ recreational and studying habits are presented in Table 3.

**Table 3:** Habits of the students with and without DED symptoms.

		Dry eye (OSDI≥13)				
		No		Yes		
		N	%	N	%	P-value
**Total number of hours spent studying per day**	0-3h	28	17.8%	26	10.8%	0.137
	3-6h	75	47.8%	123	51.2%	
	>6h	54	34.4%	91	37.9%	
**Number of hours per day spent studying books and hard copy literature**	Not using	2	1.3%	2	0.8%	0.195
	0-3h	34	21.7%	36	15.0%	
	3-6h	70	44.6%	131	54.6%	
	>6h	51	32.5%	71	29.6%	
**Number of hours per day spent studying using computers, laptops, and other VD devices**	Not using	69	43.9%	73	30.4%	**0.007**
	0-3h	85	54.1%	148	61.7%	
	3-6h	2	1.3%	15	6.3%	
	>6h	1	0.6%	4	1.7%	
**Number of hours per day spent using mobile phones, computers, laptops, and other VD devices in their free time**	Not using	0	0.0%	0	0.0%	0.911
	0-3h	75	47.8%	111	46.3%	
	3-6h	57	36.3%	87	36.3%	
	>6h	25	15.9%	42	17.5%	

A Chi square test was performed, p<0.05 was considered as statistically significant.

For further insight, univariate analysis was performed using logistic regression (Table 4). Results showed that contact lens wear was associated with a higher risk of DED (p<0.001). Initially allergies were marginally associated with DED using chi square test (p = 0.066) (Table 2). Additional logistic regression analysis demonstrated that students with allergies were more likely to have DED (p = 0.049) (Table 4). Regarding students’ studying and free time habits, the total number of hours per day spent studying did not significantly impact DED prevalence (p = 0.083) (Table 4). Also, increasing number of hours studying using hard copy literature and longer recreational VD devices use were not associated with increased likelihood of exhibiting DED (p = 0.308 and p = 0.626, respectively). The results do indicate that the time used to study using VD devices is associated with DED symptoms and that the more time was used studying with VD devices, the higher the prevalence of DED symptoms was (p = 0.014).

**Table 4:** Logistic regression analysis.

	OR	95% CI for OR	P-value
**Sex**	1.624	0.37–1.04	0.071
**Year of study**	1.100	0.97–1.24	0.123
**Contact lens wear**	3.832	2.06–7.11	**<0.001**
**Allergies**	1.637	1.00–2.67	**0.049**
**Systemic disease**	0.975	0.32–2.99	0.964
**Smoking**	1.498	0.89–2.53	0.132
**Contraceptive pills**	0.813	0.34–1.95	0.642
**Total number of hours spent studying per day**	1.753	0.93–3.31	0.083
**Number of h/day spent studying books and hard copy literature**	0.737	0.41–1.33	0.308
**Number of h/day spent studying using computers, laptops, and other VD devices**	1.616	1.10–2.37	**0.014**
**Number of h/day spent using mobile phones, computers, laptops, and other VD devices in their free time**	1.075	0.80–1.44	0.626

OR- Odds ratio; CI- Confidence Interval

## Discussion

The prevalence of symptomatic DED in this study was 60.5%, which was slightly higher than those reported in other studies including symptomatic DED with or without clinical signs in the older population [8]. Most previous studies included a population aged 40 years and older. Studies focusing on younger people, observed a relatively similar DED prevalence using the OSDI questionnaire: 50.6% among Korean university students [17], 48.1% among students in Ghana [18], 62.6% among medical students and workers at a medical university and hospitals in Dubai [19], 59.64% among undergraduate students in Brazil [20] and 50,5% among Ethiopian postgraduate students [21]. Other studies that involved student population included clinical assesment and showed a lower DED prevalence [22–24]. A recent study in Thailand observed a DED prevalence of 70.8% among medical students at Chiang Mai University. This study recognized the impact of VD devices and the psychological stress on DED among students during Covid-19 pandemic [11]. Similar studies conducted during Covid-19 pandemic reported DED prevalence from 51,8% to 71,7%, using different criteria [12–15].

In many epidemiological studies, female sex is a risk factor for DED [24–29]. The prevalence of DED in our study was higher among women than in men, but the difference did not reach statistical significance. The lack of statistical correlation between being female and DED in our study could be explained by the younger average age of the participants. The Tear Film and Ocular Surface Society, Dry Eye Workshop II indicated that the difference between sexes in symptomatic patients becomes a statistically significant risk factor only in older populations (50 years old and above) [9]. However, the Karachi Ocular Surface Study of 2433 non-clinical individuals showed no significant relationship between being female and having DED, even after excluding the younger population and analyzing only individuals aged 45 years and older [30].

We found that people using contact lenses more frequently had DED (p<0.001). Many studies among adults have shown a correlation between DED and contact lens use [10, 25]. Several studies among young adults reported similar results [11, 22, 24, 31–33]. DED occurs up to four times more frequently in the population of contact lens users than in the general population [10, 25, 31]. Contact lens use is also linked to a higher prevalence of more severe forms of symptomatic DED [34]. Contact lenses mechanically stimulate the cornea, leading to lower corneal sensitivity, relative hypoxia, and damage to nerve endings following prolonged contact lens wear. These factors affect tear film quality, integrity, and metabolic functions, reducing basal tear secretion [35]. In one study of 1844 contact lens users in Japan, symptoms of DED associated with contact lens use were present in 78.6% of the cohort. Only one-third of these cases were previously diagnosed by an ophthalmologist or an eye care provider. These findings emphasize the importance of DED screening and prevention even among young contact lens users [36].

Our logistic regression analysis results indicated that students with allergies were more likely to have DED (p = 0.049). Allergic conjunctivitis and other eye surface diseases may be associated with symptoms similar to DED, but they are considered separate clinical entities in certain studies [1, 37]. There is evidence that allergic diseases, such as vernal and atopic keratoconjunctivitis and allergic conjunctivitis, correlate with a higher risk of DED. However, this has not been proven in population studies [38–40]. These diseases should be distinguished from primary DED due to their different risk factors and therapies. The significant overlap of DED with other chronic disorders of the ocular surface, including allergies, has raised the question of whether those disorders are risk factors or contribute to the development of DED. Better understanding and recognition of the role and influence of allergies and other inflammatory diseases in developing DED could provide answers to this question in the future.

Smoking was not found to be a risk factor among the respondents of this study. In addition, studies in Singapore and Palestine found no statistically significant correlation between smoking and DED [5, 29]. However, one study in Turkey that included female smokers [41] and a meta-analysis conducted over the past ten years concerning the general population [42] indicated that smoking could be a risk factor. Therefore, further research is needed to understand and establish the role of smoking in the onset of DED.

The presence of systemic diseases and the use of contraceptive pills did not affect DED symptoms in our study. Unfortunately, we could not acquire data about the specific type of systemic disease present. Only a small fraction of students reported having a systemic disease (3.8%) or using contraceptive pills (6.8%).

The majority of the students who met the criteria for DED reported studying for 3–6 h per day. However, the total number of hours per day spent studying did not significantly impact DED prevalence (p = 0.083). One of the disadvantages of diagnosing this disorder through a questionnaire is recall bias among the participants. There is a chance that students could underestimate or overestimate their time spent on specific activities.

Our results showed an association between longer VD device use for studying and likelihood of developing DED (p = 0.014). Several studies have found a high prevalence of DED symptoms in people exposed to VD screens for work, especially among younger professionals [43–45]. A study conducted in Dubai indicated a significant association between screen time of >6 h per day and DED [19]. A recent study taken among high school students in East Java, Indonesia investigated the impact of mobile devices on evaporative DED and reported that the risk of developing evaporative DED could be induced even with a minimal use of these devices [46]. However, a study conducted in Saudi Arabia did not find a significant impact of longer electronic device use on increased computer vision syndrome symptoms among health sciences students [47]. Our results could be affected by the small sample of students who studied for more than 3 h per day using electronic sources and the possibility that the students used more than one type of literature medium (e.g., both electronic and paper). During Covid-19 pandemic several studies reported significant associtation between VD screen usage and DED among students who studied online, as well as female sex and contact lens wear [12, 13, 48]. Studies taken in Spain and Poland showed intensified DED symptoms among student population who studied online [49, 50]. In addition, one study investigated the effect of face mask usage on DED during Covid-19 pandemic. They indicated female sex, basic science years, allergy reporting, and spending more than 6h looking at screens being significantly associated with symptomatic DED, however facemask wear was not associated with DED [14]. It is still inconclusive whether the time spent studying using VD devices can exclusively increase the frequency of DED symptoms. Further research is required to determine the association between the duration of VD screen use and the development of DED symptoms. This is a potential risk factor, especially with the increasing use of VD devices for professional or personal purposes.

One strength of this study was its reasonably large sample size. The electronic survey is a more efficient and convenient means of data collection for the younger generation. In addition, information was obtained from symptomatic and asymptomatic individuals who may not seek early medical consultation. The main limitation of this study was a possible selection bias since students with DED symptoms may have been more motivated to participate in this survey than others, resulting in a higher DED prevalence. Also, another previously mentioned limitation of this type of study is recall bias.

## Conclusion

This study reported that 60.5% of the students had DED symptoms. The significant risk factors for DED were contact lens wear and allergies. Longer use of electronic devices for studying purposes was associated with a higher risk of DED. Our study is in line with a growing pool of evidence that DED symptoms are present more often and earlier in life than what was previously thought. However, additional research on the younger generation is needed to define the prevalence, incidence, and risk factors of DED, especially knowing that DED affects the quality of everyday life. Educating the youth about DED and its symptoms could potentially help with diagnosing and preventing this disease. Further research is needed to obtain a more detailed analysis of the impact of screen use on DED among the younger population and in other age groups as well.

## Supporting information

S1 Fig DED symptoms and risk factors questionnaire. (PDF)

S2 Fig Standard OSDI questionnaire and evaluation of OSDI score. (PDF)
