# Supplementary material for: Evaluation of dry eye symptoms and risk factors among medical students in Serbia
Source: PLoS One. 2022 Oct 24;17(10):e0275624. doi: 10.1371/journal.pone.0275624 (PMC9591051; doi:10.1371/journal.pone.0275624)
Supplement: S1 Fig — (PDF) [file pone.0275624.s001.pdf]

Evaluation of dry eye symptoms and risk factors among medical students

Dear collegues,

In order to determine the frequency and the impact of different risk factors on the onset and worsening of dry eye symptoms among medical student we present you with this 3 part questionnaire.

This survey is completely anonymous. Through completion of this questionnaire, you would help us analyze this problem.

We thank you for your cooperation.

If you agree with usage of given information in scientific purposes, please check the box below.

☐ Yes, I agree.

Please answer the following questions as sincere and objective as possible by choosing one of the available answers or by filling the gap beside the questions if needed.

I

1. Sex: ☐ Male

   ☐ Female
2. Age: ___________
3. Which year of study are you attending?

   ☐ 1th

   ☐ 2th

   ☐ 3th

   ☐ 4th

   ☐ 5th

   ☐ 6th
4. Do you use contact lenses?

   ☐ Yes

☐ No

5. Do you suffer from allergies?

☐ Yes

☐ No

6. Do you suffer from any systemic disease?

☐ Yes

☐ No

7. Do you smoke?

☐ Yes

☐ No

8. Do you use contraceptive pills?

☐ Yes

☐ No

II

9. How many hours per day do you study?

☐ 0-3h

☐ 3-6h

☐ More than 6h per day

10. How many hours per day do spend studying books and hard copy literature?

☐ I do not study using books and hard copy literature.

☐ 0-3h

☐ 3-6h

☐ More than 6h per day

11. How many hours per day do study using computer, laptop, and other VD devices?

☐ I do not study using computer, laptop, and other VD devices.

☐ 0-3h

☐ 3-6h

☐ More than 6h per day

12. How many hours per day do you spend using mobile phones, computers, laptops, and other VD devices in your free time?

☐ I do not use mobile phones, computers, laptops, and other VD devices in my free time.

☐ 0-3h

☐ 3-6h

☐ More than 6h per day

III

Please answer the following questions by checking the box that best represents each answer.

13. Have you experienced any of the following during the last week?

	All of the time	Most of the time	Half of the time	Some of the time	None of the time
1) Eyes that are sensitive to light?	☐	☐	☐	☐	☐
2) Eyes that feel gritty?	☐	☐	☐	☐	☐
3) Painful or sore eyes?	☐	☐	☐	☐	☐
4) Blurred vision?	☐	☐	☐	☐	☐
5) Poor vision?	☐	☐	☐	☐	☐

14. Have problems with your eyes limited you in performing any of the following during the last week?

	All of the time	Most of the time	Half of the time	Some of the time	None of the time	N/A
6)Reading?	☐	☐	☐	☐	☐	☐

7)Driving at night?	☐	☐	☐	☐	☐	☐
8)Working with a computer or bank machine (ATM)?	☐	☐	☐	☐	☐	☐
9)Watching TV?	☐	☐	☐	☐	☐	☐

15. Have your eyes felt uncomfortable in any of the following situations during the last week?

	All of the time	Most of the time	Half of the time	Some of the time	None of the time	N/A
10)Windy conditions?	☐	☐	☐	☐	☐	☐
11)Places or areas with low humidity (very dry)?	☐	☐	☐	☐	☐	☐
12)Areas that are air conditioned?	☐	☐	☐	☐	☐	☐

Thank you!
