# Supplementary material for: Evaluation of dry eye symptoms and risk factors among medical students in Serbia
Source: PLoS One. 2022 Oct 24;17(10):e0275624. doi: 10.1371/journal.pone.0275624 (PMC9591051; doi:10.1371/journal.pone.0275624)
Supplement: S2 Fig — (PDF) [file pone.0275624.s002.pdf]

Ocular Surface Disease Index© (OSDI©)[2]

Ask your patient the following 12 questions, and circle the number in the box that best represents each answer. Then, fill in boxes A, B, C, D, and E according to the instructions beside each.

HAVE YOU EXPERIENCED ANY OF THE FOLLOWING *DURING THE LAST WEEK*:

	All of the time	Most of the time	Half of the time	Some of the time	None of the time
1. Eyes that are sensitive to light?	4	3	2	1	0
2. Eyes that feel gritty?	4	3	2	1	0
3. Painful or sore eyes?	4	3	2	1	0
4. Blurred vision?	4	3	2	1	0
5. Poor vision?	4	3	2	1	0

Subtotal score for answers 1 to 5 (A)

HAVE PROBLEMS WITH YOUR EYES LIMITED YOU IN PERFORMING ANY OF THE FOLLOWING *DURING THE LAST WEEK*:

	All of the time	Most of the time	Half of the time	Some of the time	None of the time	
6. Reading?	4	3	2	1	0	N/A
7. Driving at night?	4	3	2	1	0	N/A
8. Working with a computer or bank machine (ATM)?	4	3	2	1	0	N/A
9. Watching TV?	4	3	2	1	0	N/A

Subtotal score for answers 6 to 9 (B)

HAVE YOUR EYES FELT UNCOMFORTABLE IN ANY OF THE FOLLOWING SITUATIONS *DURING THE LAST WEEK*:

	All of the time	Most of the time	Half of the time	Some of the time	None of the time	
10. Windy conditions?	4	3	2	1	0	N/A
11. Places or areas with low humidity (very dry)?	4	3	2	1	0	N/A
12. Areas that are air conditioned?	4	3	2	1	0	N/A

Subtotal score for answers 10 to 12 (C)

ADD SUBTOTALS A, B, AND C TO OBTAIN D (D = SUM OF SCORES FOR ALL QUESTIONS ANSWERED) (D)

TOTAL NUMBER OF QUESTIONS ANSWERED (DO NOT INCLUDE QUESTIONS ANSWERED N/A) (E)

Please turn over the questionnaire to calculate the patient's final OSDI© score.

Evaluating the OSDI© Score[1]

The OSDI© is assessed on a scale of 0 to 100, with higher scores representing greater disability. The index demonstrates sensitivity and specificity in distinguishing between normal subjects and patients with dry eye disease. The OSDI© is a valid and reliable instrument for measuring dry eye disease severity (normal, mild to moderate, and severe) and effect on vision-related function.

Assessing Your Patient's Dry Eye Disease[1,2]

Use your answers D and E from Side 1 to compare the sum of scores for all questions answered (D) and the number of questions answered (E) with the chart below.*
Find where your patient's score would fall. Match the corresponding shade of red to the key below to determine whether your patient's score indicates normal, mild, moderate, or severe dry eye disease.

Number of Questions Answered (E from Side 1) \ Sum of Scores for All Questions Answered (D from Side 1)	5	10	15	20	25	30	35	40	45	48
12	10.4	20.8	31.3	41.7	52.1	62.5	72.9	83.3	93.8	100.0
11	11.4	22.7	34.1	45.5	56.8	68.2	79.5	90.9	100.0	
10	12.5	25.0	37.5	50.0	62.5	75.0	87.5	100.0		
9	13.9	27.8	41.7	55.6	69.4	83.3	97.2			
8	15.6	31.3	46.9	62.5	78.1	93.8	100.0			
7	17.9	35.7	53.6	71.4	89.3	100.0				
6	20.8	41.7	62.5	83.3	100.0					
5	25.0	50.0	75.0	100.0						
4	31.3	62.5	93.8							
3	41.7	83.3								
2	62.5									
1										

Normal Mild Moderate Severe

*Values to determine dry eye disease severity calculated using the OSDI© formula:

$$\text{OSDI}^{©} = \frac{(\text{sum of scores}) \times 25}{(\text{\# of questions answered})}$$

Patient's Name: ______________________ **Date:** ____________

How long has the patient experienced dry eye? ______________________

Eye Care Professional's Comments: ______________________

____

____

____

____

____

Reference: 1. Schiffman RM, Christianson MD, Jacobsen G, Hirsch JD, Reis BL. Reliability and validity of the Ocular Surface Disease Index. *Arch Ophthalmol.* 2000;118:615-621. **2.** Data on file, Allergan, Inc.

ALLERGAN INSPIRE PHARMACEUTICALS, INC.

Tear and place in patient's chart for follow-up care on next visit.

©2004 Allergan, Inc., Irvine, CA 92612 Re-order: 4941843
